# A new pathological system for grading DCIS with improved prediction of local recurrence: results from the UKCCCR/ANZ DCIS trial

**DOI:** 10.1038/sj.bjc.6605718

**Published:** 2010-06-01

**Authors:** S E Pinder, C Duggan, I O Ellis, J Cuzick, J F Forbes, H Bishop, I S Fentiman, W D George

**Affiliations:** 1 King's College London, Division of Cancer Studies, Research Oncology, 3rd Floor, Bermondsey Wing, Guy's Hospital, Great Maze Pond, London SE1 9RT, UK; 2 Department of Epidemiology, Public Health Sciences, Fred Hutchinson Cancer Research Center, Seattle, WA, USA; 3 Histopathology Department, Molecular Medical Sciences, Nottingham University, Nottingham City Hospital, Nottingham, UK; 4 Cancer Research UK Centre for Epidemiology, Mathematics and Statistics, Queen Mary University of London, London, UK; 5 School of Medical Practice and Population Health, University of Newcastle, NSW, Australia; 6 Department of Breast Surgery, Royal Bolton Hospital, Bolton Hospitals NHS Trust, Lancs, UK; 7 Breast Unit, Guy's Hospital, London, UK; 8 University Department of Surgery, Western Infirmary, Glasgow, UK

**Keywords:** ductal carcinoma *in situ*, prognosis, histopathology

## Abstract

**Background:**

There is no consensus agreement regarding optimal management of locally excised ductal carcinoma *in situ* (DCIS) or features of greatest assistance in predicting disease behaviour. Cases in the UKCCCR/ANZ DCIS trial have been histologically reviewed to determine the features of prognostic importance.

**Method:**

A total of 72% of 1694 cases entered into the UKCCCR/ANZ DCIS trial had full pathological review. A large number of histological features were assessed, blinded to outcome and compared regarding ability to predict ipsilateral recurrence, as either DCIS or progression to invasive carcinoma.

**Results:**

Pathological features associated with ipsilateral recurrence in univariate analysis included high cytonuclear grade, larger lesion size, growth pattern, presence of necrosis or chronic inflammation, incompleteness (or uncertainty of completeness) of excision and smaller margin width. Receipt of post-operative radiotherapy was also a strong prognostic factor.

We report a novel sub-division of the large group of high-grade lesions, which enables identification of a very poor prognosis sub-group; namely, DCIS that is of high cytonuclear grade, predominantly (>50%) solid architecture, bearing extensive comedo-type necrosis (>50% of ducts). In addition, we found little difference in ipsilateral recurrence rates between low- and intermediate-grade groups. Hazard ratios for low, intermediate, high and the new, very high, grade were 0.42, 0.33, 0.62 and 1.00, respectively, for ipsilateral *in situ* or invasive recurrence.

**Conclusion:**

We present a novel pathological classification for DCIS with substantially better prognostic discrimination for ipsilateral recurrence than the classical categorisation based on cytonuclear grade alone.

Mammographic breast screening facilitates the diagnosis of ductal carcinoma *in situ* (DCIS) (Anderson *et al*, 1991) and the apparent incidence has increased as a result (Baxter *et al*, 2004). Thus, DCIS comprised 21% of screen-detected breast carcinoma in the United Kingdom in 2006–2007 (NHS Breast Screening Programme. Annual Review, 2008). In the early 1990s, three large randomised clinical trials began recruiting with the aim of assessing the safety of breast conserving surgery (BCS) for DCIS and the requirement for subsequent radiotherapy (RT) in women having wide local excision (WLE) for DCIS. The NSABP B-17 (Fisher *et al*, 1998, 1999b) and EORTC 10853 trials (Julien *et al*, 2000; Bijker *et al*, 2001, 2006), as well as the UKCCCR/ANZ DCIS trial (UK Coordinating Committee on Cancer Research Ductal Carcinoma *in situ* Working Party, 2003) recruited patients diagnosed with DCIS in the early 1990s and have already presented results comparing complete surgical excision with and without RT. Two of these trials (Fisher *et al*, 1999a; UK Coordinating Committee on Cancer Research Ductal Carcinoma *in situ* Working Party, 2003) also addressed the effect of the addition of tamoxifen to complete local excision in the management of DCIS.

The clinical management of patients with DCIS changed during the 1990s, but, despite the large reduction of local recurrence risk from RT after BCS for DCIS, only 57% of women have RT after BCS in the United Kingdom (Dodwell *et al*, 2007). There is a widespread belief that not all patients with DCIS require RT. However, the search for features that can assist in this clinical decision-making process remains unresolved (Silverstein *et al*, 1996; Sakorafas and Farley, 2003).

## Materials and methods

The UKCCCR DCIS trial was a 2 × 2 factorial randomised clinical trial comparing complete WLE alone with WLE plus RT to the residual ipsilateral breast tissue. Two further arms consisted of WLE followed by tamoxifen and WLE plus RT and tamoxifen. The dose of tamoxifen was 20 mg daily taken for 5 years. Patients receiving RT were given supervoltage treatment with opposed tangential fields that included the breast and the axillary tail. A dose of 50 Gy in 25 fractions over 5 weeks was suggested. No boost was recommended.

Patients had unilateral or bilateral DCIS detected through the UK National Health Service Breast Screening Programme, which was considered suitable for BCS. The Australian–New Zealand Breast Cancer Trials Group also participated in the trial. Patients with microinvasive carcinoma (<1 mm in maximum dimension) were eligible for inclusion. Exclusion criteria included a diagnosis of atypical ductal hyperplasia, lobular carcinoma *in situ* or Paget's disease of the nipple. Patients gave witnessed, written or verbal consent for inclusion, and local ethics approval was obtained in all centres recruiting patients.

After randomisation and treatment, patients were followed up as per local protocol. Dates of relapse (ipsilateral or contralateral), diagnosis of new, non-breast malignant disease and death (breast cancer-related or not) were recorded.

### Histological review

Representative material was sought from the surgical excision. Slides were examined by a single breast pathologist (SEP) and a large number of histological features were recorded in a database (see Table 1). Any cases that on review showed histological features, which were not sufficient for the diagnosis of DCIS, or which showed an invasive focus, were reviewed and the diagnosis confirmed by a second pathologist (IOE).

### Features recorded

The histological features of DCIS assessed are shown in Table 1. For some of these, criteria had to be specifically defined as, after literature and guideline review, it was found that no globally agreed definitions could be applied. For example, for the purposes of this study, comedo DCIS was defined specifically as (i) high-grade DCIS with (ii) central confluent necrosis and (iii) solid architectural pattern in >50% of the involved duct spaces. All these features had to be present for classification of pure comedo disease in both the traditional sub-type categorisation and for ‘Nottingham’ DCIS grade.

The original clinical protocol for the UKCCCR DCIS trial required complete excision of DCIS, but no margin width was defined. Review of completeness of excision and margin width is problematic within the auspices of a central pathology view. However, the evaluation of margin status and distance was determined on a case-by-case basis, taking into account the original histology report, number and orientation of specimens and the histological review, in which the distance (mm) to the nearest margin was measured on the histological sections received using the Vernier scale of the microscope. For the purposes of the present analysis, a distance of 1 mm or more from the nearest margin was defined as complete excision. Thus, if DCIS was stated to be 1 mm or more from the surgical margin (or completely excised) in the original report, and this was confirmed on review, excision was deemed complete. Excision was recorded as incomplete if DCIS extended to <1 mm from the margin in the original histology report (or was stated to be incompletely excised) and in the reviewed slides. Excision was coded as uncertain, when multiple pieces of unorientated tissue were received by the original pathologist or excision was stated as uncertain in original report (and no subsequent surgical procedure undertaken) or there was a discrepancy between the review and original report that could not be explained taking all features into account.

For determination of size of DCIS, the larger of the measurements of maximal dimension from either the original report or review of histological sections was recorded. If there was DCIS in first and any subsequent re-excision, or several pieces of tissue were excised bearing DCIS, the measurements were summed (recognising that this would be an approximation and would generally be an overestimate of total size). In some cases, size could not be assessed on review and was not recorded in the original histology report.

The presence and degree of associated chronic inflammation was recorded. This was typically immediately adjacent to the involved ducts spaces and some cases included nodular aggregates of lymphoid cells, with lymphoid follicle formation. In other cases, this was seen as a complete, targetoid, peri-ductal population of lymphoid cells. This was assessed in a semi-quantitative manner and scored as absent, mild, moderate or marked.

## Results

Treatment comparison analyses have been presented earlier (UK Coordinating Committee on Cancer Research Ductal Carcinoma *in situ* Working Party, 2003). Here, we present results from the histological review with the same follow-up. Between May 1990 and August 1998, 1701 patients were entered into the trial. Seven were excluded because of protocol violations (earlier malignant disease, treated by mastectomy, known to have invasive carcinoma) (*n*=1694 patients in total in the trial overall).

The number of slides submitted for pathological review varied (range 0–64 per case), with some laboratories submitting all slides, whereas others sent one representative slide or block (total number of sections 9649, mean 7.6, median 5 slides per case). On review, 33 patients were found to have earlier undiagnosed invasive carcinoma in the sections submitted (usually small and low grade) and were excluded from analysis. Twenty patients were not proven to have DCIS; in these cases, additional material was sought from the originating laboratory and reviewed, but no DCIS was identified in any of the material sent.

Sections were unavailable, or could not be retrieved, from 300 patients; these were not from any particular unit, coordinating centre or trial arm. Incomplete review data were available on a further 117 patients (e.g. the section submitted bore insufficient DCIS for a complete assessment). A total of 1224 (72.3%) patients had full data from histological review available on DCIS size, histological grade/sub-type, presence and degree of comedo-type necrosis, presence and degree of inflammation and excision status and were included in the present analyses (Table 1). Accurate review of the presence or absence of microinvasion was felt to be too error prone for meaningful analysis; some cases had the original sections submitted for review, but from other cases, new sections had been cut and submitted for re-evaluation. Nevertheless, only 16 of the 1224 cases in this series had definite microinvasion and a further 42 had ‘possible’ microinvasion in the original histology report. This feature was related to size of DCIS lesion, but not, perhaps surprisingly, to the grade of DCIS (data not shown).

Analysis for treatment arms was repeated, using the sub-set of cases with full pathological data from this review, to confirm that the results were not significantly different from the whole group analysis presented earlier (UK Coordinating Committee on Cancer Research Ductal Carcinoma *in situ* Working Party, 2003).

One hundred and fifty four of the 1224 cases reviewed (12.6%) developed recurrence of disease either DCIS or invasive carcinoma in the ipsilateral breast. Ninety nine (64%) developed recurrent DCIS and 55 invasive disease, comparable with the results in analysis of the overall trial.

Univariate analyses and distribution of features of DCIS are shown in Table 1. The DCIS was found to be of high cytonuclear grade (National Pathology Co-ordinating Group, 2005) in 74.6% of cases (*n*=913), 18.4% were of intermediate grade and only 7.0% of cases (*n*=225) were of low cytonuclear grade (*n*=86). Breakdown by Van Nuys grade (Silverstein *et al*, 1995) showed that in addition to the 913 patients (as above) with high-grade disease (74.6%), 212 had DCIS, which was non-high grade but in which necrosis was present (17.3%), and 99 patients had non-high-grade DCIS without necrosis (8.1%). Comedo-type necrosis, to a greater or lesser extent, was present in all, but 117 cases (90.4%) in this series of screen-detected DCIS.

All of the systems of grading of DCIS applied showed a significant association with recurrence of ipsilateral DCIS or invasive disease, as did the predominant growth pattern/architecture of the disease. Patients with a solid morphology as the main architectural pattern of DCIS had a 15.2% recurrence rate compared with 14.3% of those with micropapillary DCIS and only 7.3% of those with predominantly cribriform DCIS. The presence or absence of comedo-type necrosis and the presence of associated chronic inflammation was also associated with increased risk of recurrence of DCIS or progression to invasive cancer in the ipsilateral breast (Table 1).

Intragrade analysis showed that the pure comedo-type cases (*n*=483, 39.5%) fared particularly poorly. This grading system recognised a large group of cases in this trial as ‘non-pure comedo’ type disease (51.0%).

**Figure 1:**
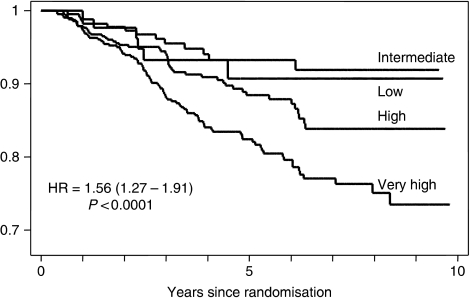
Recurrence of ipsilateral DCIS or invasive carcinoma by new grading system for DCIS.

**Figure 2:**
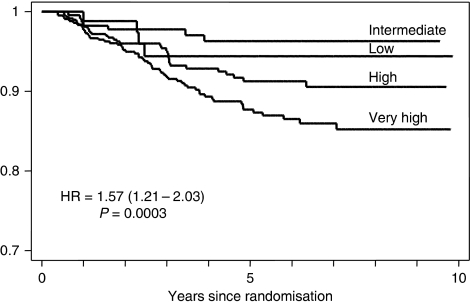
Recurrence of ipsilateral DCIS by new grading system for DCIS.

**Figure 3:**
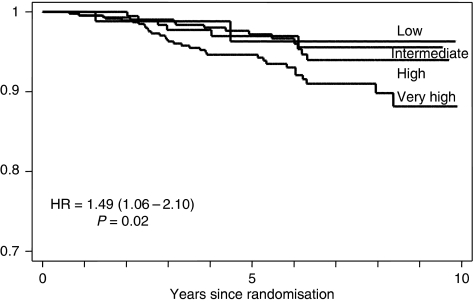
Recurrence of ipsilateral invasive carcinoma by new grading system for DCIS.

Re-analysis of cytonuclear grade with the inclusion of an additional particularly aggressive ‘pure comedo’ group was, therefore, undertaken. Using the definitions applied in this review, this latter group had (i) high-cytonuclear-grade DCIS, (ii) >50% solid architecture and (iii) >50% of the ducts bore central confluent comedo-type necrosis. This resulted in a new four-tiered system: low (low cytonuclear grade (7.0% cases)), intermediate (intermediate cytonuclear grade (18.4% of cases), high (high cytonuclear grade, but not pure comedo (i.e. not predominantly solid architecture or <50% ducts bore necrosis) (35.1%)) and very high (high-grade DCIS of >50% solid architecture and >50% ducts bearing comedo-type necrosis (39.5% of cases)). This novel classification system showed a strong relationship with development of ipsilateral recurrence (Table 2a), both overall and separately for DCIS and invasive disease (Figures 1, 2 and 3). This new classification system, retained significance across the range of sizes of disease (although numbers in the groups are by necessity smaller). Even, for example, for those lesions 10–19 mm in size, the new system could distinguish a group of patients with very high-grade disease who had a significantly higher risk of developing ipsilateral recurrence of DCIS or invasive carcinoma over the remainder of those with cytonuclear high-grade DCIS (Table 2b).

The maximum dimension of DCIS (the larger size from either original histology report or histological review) ranged from 0.5 to 82 mm (median 14.5 mm). There were 368 patients with DCIS <10 mm in size (30.1%), 575 (46.9%) had DCIS measuring 10–19 mm and 268 patients (21.9%) had lesions ⩾20 mm. The total size of DCIS could not be accurately determined in 13 patients. Univariate analysis showed that larger DCIS size (⩾20 mm) was associated with a higher risk of ipsilateral recurrence.

Despite the inclusion criteria requiring ‘complete excision’, this was not quantified in the original trial protocol. Defining complete excision as 1 mm or more distance to the nearest margin for this analysis, it was found that 196 patients (16.0%) had incompletely excised disease. In 182 patients, the completeness of excision was uncertain (14.9%). The majority (69.1%, *n*=846) had DCIS that was completely excised ⩾1 mm; this was associated with a lower risk of ipsilateral recurrence of disease (DCIS or invasive). No association was seen between completeness of excision and DCIS size, or with grade of DCIS (data not shown).

An absolute measurement of the distance to the margins in mm was available in 637 cases, from the original report or the histological review. The range of distance to the surgical margins was 0–25 mm, the median was 1.5 mm and the mean 2.8 mm. Some cases of DCIS were assessed as excision ‘uncertain’ as a categorical variable, as described above, for example when there was disagreement between the original histology report and review. For this reason, there was a greater number of cases with a margin measurement of <1 mm (*n*=269; 42.2%) than were classified as incompletely excised, as described above (*n*=196). Of the group with assessable margin width, 214 (33.6%) had disease between 1 and <5 mm from the margin and 154 (24.2%) had disease 5 mm or more from an inked radial margin of excision. Only 29 patients (4.5%) had disease >10 mm from the margin. Analysis of margin distance (sub-grouped either as <1 mm; 1 to <2 mm; 2 to <5 mm; 5 mm or more) showed that the distance of disease to the margin was a significant predictor of ipsilateral recurrence of disease (DCIS or invasive) (Table 3). The DCIS excised by ⩾5 mm had approximately half the risk of ipsilateral recurrence compared with DCIS excised by <1 mm.

The presence of chronic inflammation was related to recurrence of ipsilateral disease (Table 1). Of note, this was particularly associated with an increase in progression to ipsilateral invasive disease recurrence rather than recurrent DCIS. Those cases without chronic inflammation had a hazard ratio (HR) for ipsilateral invasive disease recurrence of 0.27 compared with cases in which an inflammatory cell infiltrate was identified. For DCIS recurrence, in the absence of chronic inflammation, the HR was 0.58. Of note, this feature was very commonly present to some degree (76% of cases) and was related to the grade of DCIS (seen in 21%, 55% and 86% of cytonuclear grades 1, 2 and 3, respectively, *χ*^2^=250.0, *P*<0.001), but was independent of grade of DCIS (and also the new classification system) in multivariate analysis (Table 4).

Multivariate analysis including the new grading four-tier system for DCIS, the receipt of RT, tumour size and completeness of excision is shown in Table 4; the HR of the new very high grade was 2.77 for any ipsilateral recurrence when compared with low- and intermediate-grade DCIS. Multivariate analysis also confirmed that RT reduced the risk of ipsilateral DCIS and invasive recurrence by approximately one-third (HR=0.34, CI=0.23–0.52) in this large sub-set of pathologically reviewed cases from the main trial. Increasing DCIS size was also of independent significance.

## Discussion

Many of the cases did not have what would now be considered the minimum dataset of features for DCIS reported in the original laboratory (Lester *et al*, 2009); all the histological features presented here are derived from the central histopathology review. Such review of any large multicentre trial is fraught with difficulties, including the retrieval of histological sections from a large number of different laboratories. This is particularly problematic for trials of DCIS, as more than one surgical procedure is more frequently undertaken than for invasive breast carcinoma. In total, 72.3% of the cases had material reviewed.

The determination of completeness of excision of DCIS can be difficult for the primary reporting pathologist, but especially in a central review; for this reason, this feature has not been included in the analysis in many papers (de Mascarel *et al*, 2000; Ottensen *et al*, 2000). We report details of margin status and distance to margins in the present analysis, but would note that the evaluation of this feature and, in particularly, the measurement of distance to margins cannot be considered precise. Despite this, both completeness of excision as a categorical variable and the distance to margins were found to be of independent significance in predicting for ipsilateral recurrence of disease, highlighting again the significance of this feature in clinical management of DCIS.

No defined margin distance was required for inclusion into this randomised clinical trial. Pragmatically, complete excision was defined as ⩾1 mm in this pathological review analysis; many will claim this cutoff is too small. The margin width desirable for adequate treatment of pure DCIS remains a topic of major controversy with some of the opinion that the margin should simply be ‘tumour free’ (Fisher *et al*, 1993). It has been suggested that a 2 mm margin is sufficient if RT is also given (Kell and Morrow, 2005), but others recommend that a larger width of tumour-free tissue is appropriate and have shown an increasing HR for local recurrence with decreasing margin clearance in patients with DCIS treated by excision alone (MacDonald *et al*, 2005). We have found that complete excision (⩾1 mm) was associated with a lower risk of local recurrence of ipsilateral disease, but the present analysis does not unequivocally assist in distinguishing what margin width is optimal (Table 3).

The presence of chronic inflammation was found to be a predictor of a higher risk of ipsilateral recurrence of invasive disease, but not of recurrent DCIS. This was related to, but statistically independent of, cytonuclear grade. The presence of chronic inflammation may be associated with invasive and angiogenic factors, a local milieu, which facilitates invasion through the basement membrane. An association with the development of invasive carcinoma thus seems plausible, but the association with recurrence independent of features such as completeness of excision is difficult to explain and clearly requires further investigation. Further evaluation of the nature of the chronic inflammation was not possible using the routinely stained sections in this review, but seems warranted.

Patient age was significantly associated with disease recurrence in univariate analyses (*P*=0.05), but this was not retained in the multivariate model. However, the majority of patients recruited to the trial had DCIS detected through the UK National Health Service Breast Screening Programme available to all women aged 50–64 years (at that time). Thus, only 92 of the 1224 women in this analysis were ⩽50 years of age, so comment cannot be made regarding young patient age and risk of recurrence from this clinical trial.

The distribution of cytonuclear grade of the DCIS in this UK trial is different to that in other series such as the EORTC 10853 trial; in the latter study, only 40% of cases were poorly differentiated, 28% were moderately differentiated and 32% were well differentiated (Bijker *et al*, 2001). In comparison, the proportion of DCIS of high grade in the present series is large (74.6%). Although this is likely, at least in part, to be a reflection of the era in which the patients were recruited to the trial, high-grade DCIS remains the commonest form identified in the United Kingdom. In the Sloane Project/UK audit of screen-detected DCIS, 62% of 1101 DCIS, in which grade was recorded, were of high grade (Thomas *et al*, 2008). Although difficulties regarding the reproducibility of grade of DCIS between pathologists have been described (Ellis *et al*, 2006), in the present review, all cases were examined by one pathologist, reducing this element of variation.

DCIS is a heterogeneous disease morphologically, immunohistochemically and genetically (Patchefsky *et al*, 1989; Bobrow *et al*, 1994; Meijnen *et al*, 2008; Vincent-Salomon *et al*, 2008) and sub-typing can identify lesions with differing risks of progression and recurrence. In this study, risk of ipsilateral recurrence was related to sub-type of disease, using many of the grading systems described (Holland *et al*, 1994; Poller *et al*, 1994; Silverstein *et al*, 1995; National Pathology Co-ordinating Group, 2005), as shown in Table 1, or indeed the older historical method of sub-typing based on architecture and cell size. The presence of comedo-type necrosis also predicted outcome, as shown in other studies (Fisher *et al*, 1995, 1999b; Silverstein *et al*, 1996). Growth pattern/architecture also reflected risk of recurrence, as shown in the EORTC DCIS trial (Bijker *et al*, 2001). In the present large series reviewed by a single breast pathologist, many of the earlier described prognostic features of DCIS showed a relationship with risk of ipsilateral recurrence of disease; the question then remains as to which of these features is/are most valuable?

In this study, we have identified a group of patients with a particularly poor outcome, which has not been shown earlier. The novel finding in this series is that those women who had DCIS not only of high nuclear grade, but also of pure (>50%) solid architecture with extensive necrosis (in >50% of ducts) had a significantly worse outcome than those with a high cytonuclear grade alone. Thus, the division of DCIS into low-, intermediate- and high-grade forms, as applied in present systems of classification (National Pathology Co-ordinating Group, 2005; Lester *et al*, 2009), may not be optimal. The relatively small proportion of disease of low or intermediate grade, and the few events in these groups, makes it difficult to draw definitive conclusions from the present series, but patients with low- or intermediate-grade DCIS fared similarly well. Of note, low- and intermediate-grade DCIS have also been shown to have similar immunohistochemical profiles, distinct from that of high-grade disease (Meijnen *et al*, 2008).

The system we describe suggests that DCIS is better classified by an alternative three group system: (i) a group of low- and intermediate-cytonuclear-grade disease, (ii) high-nuclear-grade DCIS of non-solid architecture or with <50% ducts bearing necrosis and (iii) high-nuclear-grade DCIS with extensive confluent comedo-type necrosis (>50% ducts) and with solid architecture. These groups have ipsilateral recurrence rates of DCIS or invasive disease of 6.1%, 10.9% and 18.2%, respectively. Although this proposed system of classification clearly requires further validation in other series, it may potentially aid in the recognition of more clinically relevant groups of patients with DCIS, namely women with low-risk disease who may not require further adjuvant therapy to the breast and a group with a particular high risk of recurrence who may benefit from maximal local therapy.

## Figures and Tables

**Table 1:** Univariate analysis – recurrence of ipsilateral DCIS or invasive disease

**Feature**	**Categories**	**No. cases**	**No. events**	**HR (95% CI)**	***χ*^2^ for trend *P-*value**
Cytonuclear grade (National Pathology Co-ordinating Group, 2005)	Low	86	6	0.51 (0.22–1.15)	14.58
	Intermediate	225	13	0.41 (0.23–0.72)	*P*=0.0007
	High	913	135	1.00^a^	
Traditional/historical nomenclature	Small cell micropapillary	17	3	1.32 (0.42–4.16)	39.34
	Small cell cribriform	86	0	0 (0.00–0.20)	*P*<0.0001
	Small cell solid	31	3	0.53 (0.17–1.68)	
	Small cell mixed	65	7	0.63 (0.29–1.37)	
	Large cell mixed	298	31	0.60 (0.40–0.90)	
	Large cell cribriform	130	10	0.41 (0.21–0.80)	
	Large cell solid	86	11	0.75 (0.40–1.40)	
	Comedo	483	88	1.00^a^	
	Papillary	28	1	0.20 (0.03–1.45)	
Van Nuys grade (Silverstein *et al*, 1995)	Non-high grade without necrosis	99	5	0.39 (0.16–0.94)	14.58
	Non-high grade with necrosis	212	14	0.45 (0.26–0.78)	*P*=0.0007
	High	913	135	1.00^a^	
Nottingham grade (Poller *et al*, 1994)	DCIS without necrosis	117	6	0.58 (0.25–1.35)	19.38
	Non-pure comedo	624	88	1.00^a^	*P*=0.0001
	Comedo	483	60	1.85 (1.33–2.57)	
Differentiation (Holland *et al*, 1994)	Well differentiated	90	6	0.38 (0.22–0.66)	18.09
	Moderately differentiated	248	14	0.47 (0.21–1.07)	*P*=0.0001
	Poorly differentiated	886	134	1.00^a^	
Main architecture	Solid	731	111	1.00^a^	15.14
	Cribriform	372	27	0.47 (0.31–0.71)	*P*=0.002
	Micropapillary	91	13	0.97 (0.54–1.71)	
	Papillary	30	3	0.50 (0.20–2.02)	
Necrosis	Confluent comedo necrosis present	1107	147	1.00^a^	4.07
	Confluent comedo necrosis not present	117	7	0.50 (0.23–1.06)	*P*=0.04
Age	>50 years^a^	1132	137	1.00^a^	3.74
	⩽50 years	92	17	1.71 (1.03–2.82)	*P*=0.05
Chronic inflammation associated with DCIS	Present	924	135	1.00^a^	12.78
	Not present	296	19	0.45 (0.27–0.73)	*P*<0.0001
Histological calcification	Present	1089	135	1.00^a^	1.11
	Not present	129	19	1.31 (0.81–2.11)	NS
Tumour size	0–0.9 cm	368	27	0.55 (0.35–0.85)	20.04
	1.0–1.9 cm	575	77	1.00^a^	*P*<0.0001
	>2.0 cm	268	49	1.55 (1.08–2.22)	
Excision	Complete (1 mm or more from margin)	846	91	1.00^a^	7.77
	Incomplete (DCIS<1 mm from margin)	196	31	1.50 (1.00–2.26)	*P*=0.02
	Uncertain	182	32	1.67 (1.12–2.50)	

Abbreviations: CI=confidence interval; DCIS=ductal carcinoma *in situ*; HR=hazard ratio.

a Baseline category. Cases with missing values for a variable have been excluded from that analysis.

**Table 2a:** New grading system – recurrence of ipsilateral DCIS or invasive carcinoma

**Feature**	**Category**	**No. cases**	**No. events**	**HR (95% CI)**	***χ*^2^ for trend, *P-*value**
*Recurrence of ipsilateral DCIS or invasive*					
Grading system, four tier	Low	86	6	0.42 (0.18–0.95)	22.11
	Intermediate	225	13	0.33 (0.19–0.60)	*P*=0.0001
	High	430	47	0.62 (0.43–0.88)	
	Very high	483	88	1.00^a^	
New grade, three tier	Low/intermediate	311	19	0.36 (0.22–0.58)	21.91
	High	430	47	0.62 (0.43–0.88)	*P*=0.0001
	Very high	483	88	1.00^a^	
					
*Recurrence of ipsilateral DCIS*					
Grading system, four tier	Low	86	4	0.44 (0.15–1.20)	15.42
	Intermediate	225	7	0.28 (0.12–0.62)	*P*=0.002
	High	430	32	0.66 (0.43–1.02)	
	Very high	483	56	1.00	
New grade, three tier	Low/intermediate	311	11	0.32 (0.16–0.61)	14.97
	High	430	32	0.66 (0.43–1.02)	*P*=0.0006
	Very high	483	56	1.00	
					
*Recurrence of ipsilateral invasive*					
Grading system, four tier	Low	86	2	0.41 (0.10–1.72)	6.67
	Intermediate	225	6	0.46 (0.19–1.11)	*P*=0.08
	High	430	14	0.53 (0.28–0.99)	
	Very high	483	32	1.00	
New grade, three tier	Low/intermediate	311	8	0.45 (0.21–0.98)	6.65
	High	430	14	0.53 (0.28–0.99)	*P*=0.04
	Very high	483	32	1.00	

Abbreviations: CI=confidence interval; DCIS=ductal carcinoma *in situ*; HR=hazard ratio.

a Baseline category. *NB*: There is one ipsilateral event for which it is unknown whether the recurrence was DCIS or invasive.

**Table 2b:** New grading system for size groups – recurrence of ipsilateral DCIS or invasive carcinoma

**Feature**	**Category**	**No. cases**	**No. events**	**HR (95% CI)**	***χ*^2^ for trend, *P-*value**
*Tumour size <9 mm*					
New grade, three tier	Low/intermediate	132	3	0.21 (0.05–0.71)	8.57
	High	108	9	0.75 (0.32 – 1.71)	*P*=0.011
	Very High	128	15	1.00^a^	
*Tumour size 10–19 mm*					
New grade, three tier	Low/intermediate	116	9	0.43 (0.21–0.90)	8.77
	High	222	23	0.55 (0.33–0.91)	*P*=0.01
	Very high	237	45	1.00^a^	
*Tumour size >20 mm*					
New grade, three tier	Low/intermediate	62	7	0.46 (0.31–1.15)	4.65
	High	94	14	0.46 (0.20–1.06)	*P*=0.09
	Very high	112	28	1.00^a^	

Abbreviations: CI=confidence interval; DCIS=ductal carcinoma *in situ*; HR=hazard ratio.

a Baseline category.

**Table 3:** Margin distance as a predictor of outcome

**End point**	**Distance to margin^a^**	**No. cases**	**No. events**	**HR (95% CI)**	***χ*^2^ for trend, *P-*value**
Ipsilateral DCIS or invasive recurrence	0–<1 mm	269	47	1.00^b^	4.93
	⩾1–<2 mm	71	6	0.47 (0.20–1.11)	*P=0.03*
	⩾2–<5 mm	140	20	0.88 (0.52–1.48)	
	⩾5 mm	157	13	0.46 (0.25–0.86)	
Ipsilateral DCIS or invasive recurrence	0–<1 mm	269	47	1.00	*4.98*
	⩾1 mm	368	39	0.61 (0.41–0.94)	*0.03*

Abbreviations: CI=confidence interval; DCIS=ductal carcinoma *in situ*; HR=hazard ratio.

a Baseline category.

b The smaller of the measurement (i) from the original histology report (if noted) or (ii) from the histological review of slides was used for analysis, unless there was a clear discrepancy when the margin distance was recorded as not assessable.

**Table 4:** Multivariate analysis for all ipsilateral recurrent events and for recurrence of ipsilateral invasive disease

	**Recurrence of all ipsilateral disease (DCIS or invasive)**			**Recurrence of ipsilateral invasive carcinoma**		
**Covariate**	**No. cases**	**HR**	**95% CI**	**No. cases**	**HR**	**95% CI**
*New grading system*						
Low and intermediate	310	1^a^		309	1^a^	
High	424	1.69	0.99–2.89	423	0.86	0.35–2.11
Very high	477	2.77	1.69–4.57	476	1.46	0.65–3.31
						
*XRT received*						
No XRT	776	1^a^		774	1^a^	
XRT	435	0.34	0.23–0.52	434	0.52	0.27–0.98
						
*Tumour size*						
0–0.9 cm	368	1^a^		366	1^a^	
1.0–1.9 cm	575	1.67	1.07–2.59	574	1.89	0.89–3.99
⩾2 cm	268	2.67	1.66–4.30	268	1.89	0.80–4.49
						
*Excision*						
Complete	838	1^a^		835	1^a^	
Incomplete	192	1.47	0.98–2.22	192	1.01	0.46–2.21
Uncertain	181	1.82	1.21–2.73	181	2.35	1.27–4.37
						
*Inflammation*						
Absent				294	1^a^	
Present				914	3.11	1.06–9.13

Abbreviations: CI=confidence interval; DCIS=ductal carcinoma *in situ*; HR=hazard ratio; XRT=radiotherapy.

a Baseline category.
